# Knowledge, attitudes, and practices of seasonal influenza vaccination in postpartum women, Honduras

**DOI:** 10.1371/journal.pone.0246385

**Published:** 2021-02-11

**Authors:** Zachary J. Madewell, Rafael Chacón-Fuentes, Jorge Jara, Homer Mejía-Santos, Ida-Berenice Molina, Juan Pablo Alvis-Estrada, Rosa Coello-Licona, Belinda Montejo

**Affiliations:** 1 Centro de Estudios en Salud, Universidad del Valle de Guatemala, Guatemala City, Guatemala; 2 Unidad de Vigilancia de la Salud, Secretaría de Salud de Honduras, Tegucigalpa, Honduras; 3 Programa Ampliado de Inmunizaciones, Secretaría de Salud de Honduras, Tegucigalpa, Honduras; 4 Vigilancia Epidemiológica, Instituto Hondureño de Seguridad Social, Tegucigalpa, Honduras; Waikato Institute of Technology, NEW ZEALAND

## Abstract

**Background:**

Influenza during pregnancy may cause serious neonatal outcomes including stillbirth, fetal distress, preterm birth, congenital abnormalities, and stunted growth. Pregnant women are the highest priority group for seasonal influenza vaccination, but low coverage has been repeatedly reported in this population. Understanding reasons for and for not receiving the seasonal influenza vaccine is needed to design communication strategies to increase vaccination coverage. This study aimed to describe knowledge, attitudes, and practices (KAP) of seasonal influenza vaccination among women giving birth in public maternity hospitals in Honduras.

**Methods:**

From August 20–October 8, 2018, we conducted a cross-sectional KAP survey regarding seasonal influenza vaccinations to a sample of postpartum women who gave birth in maternity hospitals and clinics from the Ministry of Health of Honduras and Honduran Social Security Institute. We reported frequency distributions for demographics, KAP of influenza vaccine, and vaccination coverage. We used logistic regression to analyze unadjusted and adjusted associations between sociodemographic characteristics and influenza vaccination.

**Results:**

We surveyed 842 postpartum women in 17 healthcare facilities. Of 534 postpartum women with term pregnancy and verified vaccinations, 417 (78.1%; 95% CI: 74.6–81.6%) were vaccinated for influenza. Factors associated with verified influenza vaccination included receipt of vaccination recommendations by a healthcare worker during prenatal check-ups (aOR: 16.46; 95% CI: 9.73–27.85), concurrent chronic disease (aOR: 5.00; 95% CI: 1.25–20.07), and influenza vaccination of other children in the household (aOR: 2.28; 95% CI: 1.19–4.39). The most cited reasons for vaccination were perceived benefits for both mother and infant and easy access. Reasons for non-vaccination were: vaccine was not offered and fear of side effects, harm to the infant, and needles or pain caused by injection.

**Conclusion:**

Influenza vaccination was well received among postpartum women in Honduras. Increasing clinician recommendations for vaccination and assuring the vaccine is readily available to women during prenatal visits may increase vaccination rates.

## Introduction

Influenza is a highly infectious respiratory disease that causes fever, headache, musculoskeletal pain, malaise, sore throat, and cough [1]. It can also lead to serious and life-threatening complications, including upper respiratory tract infections and pneumonia [1]. The World Health Organization (WHO) estimates that 5–10% of adults and 20–30% of children contract influenza each year [2], resulting in 3–5 million cases of serious illness and 290,000–650,000 deaths worldwide [2, 3].

WHO Strategic Advisory Group of Experts (SAGE) on Immunization considers pregnant women the highest priority group for seasonal influenza vaccination and recommends vaccination at any stage of pregnancy [2]. Pregnant women who contract influenza, particularly those with underlying medical conditions, are at increased risk for hospitalization and complications compared to non-pregnant women [4, 5]. This may result in part from adverse immunologic, pulmonary, and cardiovascular effects. Risk of serious morbidity and complications increase with gestational age, especially during the third trimester of pregnancy [4, 5]. Influenza during pregnancy may also cause serious neonatal outcomes including stillbirth, preterm birth, fetal distress, congenital abnormalities, and stunted growth [6]. Influenza vaccination during pregnancy effectively prevents influenza in pregnant women and their infants [7, 8] and may protect newborns and infants <6 months of age, who are ineligible for the vaccine [4, 9]. Following recommendations from SAGE and the Pan American Health Organization’s (PAHO) Technical Advisory Group, 39 countries or territories in the Americas initiated seasonal influenza vaccination among high risk groups including 31 for pregnant women [10].

In Central America, influenza epidemics typically begin in June and last five months with influenza A (H1N1 and H3N2) as the predominant strains in the region [11]. In Central American countries, people of high risk groups are vaccinated for seasonal influenza during annual vaccination campaigns. With the exception of Belize and Guatemala, the composition vaccine for the Southern Hemisphere is used in these campaigns, which are conducted from April to June each year [12, 13]. In Central American countries, pregnant women may be vaccinated for influenza at any stage of their pregnancy [13, 14].

The seasonal influenza vaccine is effective at preventing influenza and reducing influenza-related illnesses [15, 16], but is not used uniformly in Central American countries. In 2018, seasonal influenza vaccination coverage among pregnant women ranged from 48% in Belize to 91% in Nicaragua [17]. Furthermore, although pregnant women are the highest priority group for seasonal influenza vaccination, low coverage has been repeatedly reported in the literature about this population [18, 19]. Reasons cited in other studies for non-vaccination include underestimating risks of complications from influenza, concerns about the safety and efficacy of vaccination during pregnancy, and not receiving a firm recommendation to get vaccinated from obstetric healthcare providers [18, 20]. Moreover, since vaccination is mainly carried out during campaigns, there are segments of the population that do not have systematic access to vaccines (e.g., women who start their pregnancy after the end of the vaccination campaign, but end their pregnancy before the onset of the vaccination campaign the following year). Understanding reasons for and for not receiving the seasonal influenza vaccine is prerequisite for the design of effective vaccination strategies.

This study was set in Honduras, where the annual influenza-associated hospitalizations (66.2 per 100,000; 95% CI: 20.0–197.8) and deaths (0.7 per 100,000; 95% CI: 0.3–1.2) were lower than those of Latin America and the Caribbean (hospitalizations: 74.6 per 100,000; 95% CI: 30.5–176.8; deaths: 2.3 per 100,000; 95% CI: 1.6–3.2) in 2017 [21]. Influenza-associated lower respiratory tract infections in Honduras (645.9 per 100,000; 95% CI: 430.9–925.5) were higher than those of Latin America and the Caribbean (457.7 per 100,000; 95% CI: 321.6–623.4) [21]. For Honduras compared to Latin America and the Caribbean, the average fertility rate is 2.46 and 2.03 births per woman [22], mortality rate for infants is 22.8 and 14.8 per 1,000 live births [23], mortality rate for children <5 years is 17.4 and 16.7 per 1,000 live births [23], life expectancy for women is 77.4 and 78.6 years [22], and mortality rate for female adults is 115.5 and 92.7 per 1,000 female adults [22], respectively. In 2013, 25% of Honduran infants were born to adolescent mothers, the second highest teenage pregnancy rate in Latin America, after Nicaragua [24].

Honduras initiated seasonal influenza vaccination campaigns for high risk groups in 2003, including pregnant women at any stage, children 6 months to five years of age, adults >60 years of age, people with chronic diseases, healthcare workers, and poultry farm workers [25]. Seasonal influenza vaccination has been recommended for pregnant women of any gestational age in Honduras since 2015 [26].

### Honduras and its healthcare system

Honduras has an area of 112,492 km^2^ and is divided administratively into 18 departments, which are political subdivisions similar to states. Departments vary in size from 236 kilometers^2^ for Islas de la Bahía to 24,057 km^2^ for Olancho [27]. The population of Honduras was 9,746,000 people (2019), of which 57.7% reside in urban areas and 50.0% are women, and population density was 85.7 per km^2^ (2018) [28].

The healthcare sector in Honduras consists of public and private subsectors. The public healthcare sector is composed of the Ministry of Health of Honduras (SESAL) and Honduran Social Security Institute (IHSS). SESAL provides healthcare services to the entire Honduran population, whereas IHSS serves contributing workers and employers in its service delivery regime. SESAL and IHSS serve 60% and 12% of the population, respectively [29]. SESAL includes seven national hospitals, six regional hospitals, 16 area hospitals, and 1,606 first-level outpatient facilities [29]. IHSS includes two hospitals and 11 outpatient healthcare facilities [29]. Approximately 17% of the population does not have access to healthcare services [30]. Most healthcare facilities are located in urban areas, which presents access challenges for indigenous and rural populations [31]. In 2016, national public health expenditure was 3.9% of Honduras’s GDP, similar to that of Latin America and the Caribbean (3.7%) [23].

We aim to describe the knowledge, attitudes, and practices (KAP) of vaccination for seasonal influenza among women giving birth in maternity hospitals in Honduras. The results of this study may be used to support the orientation of communication and education strategies aimed at facilitating access to seasonal influenza vaccination for pregnant women.

## Materials and methods

### Study design

We conducted a cross-sectional KAP survey regarding seasonal influenza vaccinations to a sample of postpartum women who gave birth in maternity hospitals and clinics, from both SESAL and IHSS.

### Setting

**Fig 1:**
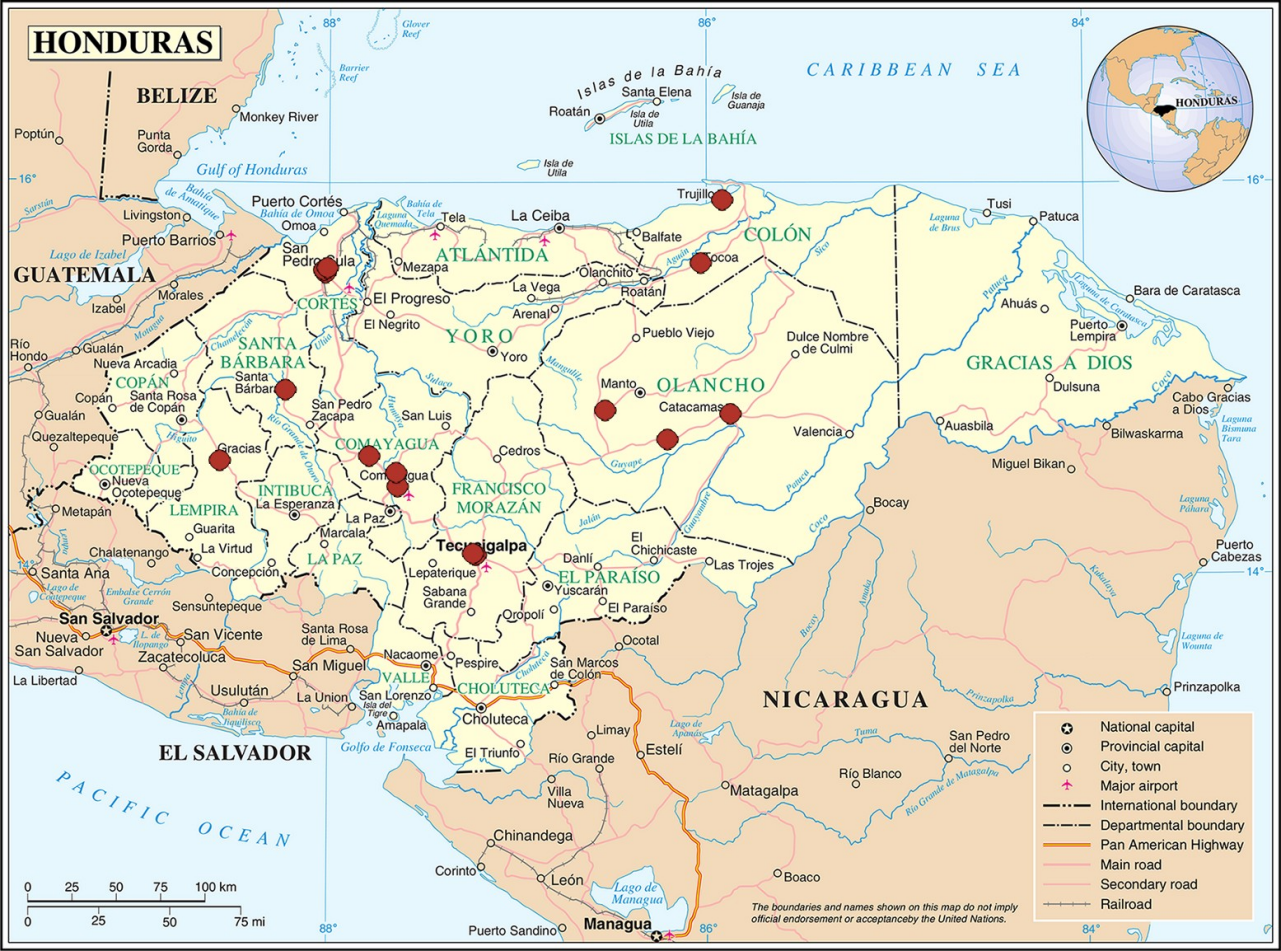
Location of the 17 maternity hospitals and clinics from Ministry of Health of Honduras and Honduran Social Security Institute, study of knowledge, attitudes and practices of seasonal influenza vaccination, postpartum women, Honduras, 2018. Source: This map is a public domain file provided by the Central Intelligence Agency’s World Factbook, https://www.cia.gov/library/publications/the-world-factbook/.

Data was collected from SESAL and IHSS hospitals in eight of the 18 departments of Honduras (Fig 1).

### Participants

To calculate sample size, we used the lowest administrative vaccination coverage for influenza among pregnant women in Central American countries reported by PAHO in 2015 as a key indicator: 51% [32]. For the reference population, we used birth projections from June 1 to July 31, 2018: 25,393 births [33]. We used a design effect of two, corresponding to the two stages of sampling described below. We also used a replacement rate of 10% based on previous experiences from other KAP studies of postpartum women in Costa Rica and Guatemala [34, 35]. Using 5% accuracy and a 95% confidence interval, we calculated a sample size of 841 postpartum women (S1 File).

We used probabilistic, two-stage, stratified and conglomerate sampling to select samples of postpartum women in hospitals or clinics of SESAL and IHSS. Stratification was based on hospital or clinic locations (West, Northeast, Central). In stage one, we identified conglomerates (hospitals, clinics) in each stratum by probability proportional to the number of deliveries in each hospital or clinic. In stage two, we invited all postpartum women in each cluster who were available between August 20 and October 8 to participate. In selected maternity hospitals and clinics, we coordinated with ward nurses to identify potential participants in the immediate postpartum period. For each potential participant identified, we first described the study, obtained informed consent, and verbally administered a survey. Excluded participants were those who could not respond to interview questions because of their clinical condition, nonresidents of Honduras, and women who remained outside Honduras throughout their pregnancy, up to seven days before delivery.

### Measures

We adapted a close-ended survey from the Centers for Disease Control and Prevention (CDC) influenza survey [36] and previous experience from other KAP studies of postpartum women in Costa Rica and Guatemala [34, 35]. Both Spanish and English surveys are available from the CDC [36]. The survey was modified following an evaluation of technical detail and cultural appropriateness by personnel from SESAL and IHSS, and by the Institutional Review Boards of Universidad del Valle de Guatemala (UVG) and Universidad Nacional Autónoma de Honduras (UNAH). We pilot-tested the survey and informed consent with ten postpartum women at San Felipe General Hospital in Tegucigalpa four weeks before study implementation. Minimal modifications were made to the survey following feedback provided by the participants. The finalized survey had 41 items including demographics (age, department of residence, race, education, occupation, marital status, concurrent chronic disease, gestational age, number of children in the household, vaccination status of other children in household, number of prenatal visits, healthcare worker recommended vaccine, distance to nearest vaccination site), knowledge of benefits and risks of influenza vaccination, influenza vaccination status, reasons for and for not receiving influenza vaccination, perceived risk of influenza, and clinical manifestations following vaccination (S1 Survey).

### Data collection

We conducted surveys from August 20 to October 8, 2018, three months after the launch of the influenza vaccination campaign of Honduras on May 14, 2018. Surveys were administered at least 20 hours postpartum in the maternity hospitals and clinics from which the postpartum women were recruited. We conducted surveys verbally in Spanish and collected data with tablets, using the Research Data Management Center application (Open Data Kit ODK JAVA). Interviewers were healthcare professionals specifically trained on interview methodology and use of tablets to record responses. There were no open-ended questions to minimize interviewer bias. We reviewed vaccination cards and medical records from the Latin American Perinatology Center to verify influenza vaccination status.

### Ethics statement

This study was approved by the Research Ethics Committee of UVG (Protocol number 173-10-2017), Bioethics Committee of UNAH (study code 2018011), and Teaching and Research Department of IHSS. We obtained written informed consent for adults ages ≥18 years and emancipated minors. We obtained verbal assent from non-emancipated minors and informed consent from their parents or legal guardians.

### Statistical analysis

We reported frequency distributions for demographic variables (age group, department, race, education, occupation, marital status, concurrent chronic disease, gestational age, number of children in the household, vaccination status of other children in household, number of prenatal visits, healthcare worker recommended vaccine, health system, SESAL/IHSS medical setting, distance to nearest vaccination site, self-reported and verified vaccination status in 2018). We reported frequencies, proportions, and 95% confidence intervals (CI) of knowledge of influenza vaccination, reasons for and for not receiving vaccination, and clinical manifestations within seven days of vaccination.

We used logistic regression to analyze associations between exposures of interest (age group, department, education, occupation, marital status, gestational age, number of children in household, vaccination status of other children in household, number of prenatal visits, concurrent chronic disease, received vaccination recommendation from a healthcare worker, health system, medical setting, distance to nearest vaccination site), and 1) verified and 2) self-reported influenza vaccination status. Participants self-reported their vaccination status, which we subsequently verified with vaccination cards and medical records from the Latin American Perinatology Center to improve veracity of our findings. These analyses excluded those who did not provide complete demographic information. Analyses of verified influenza vaccinations excluded participants with unverified vaccinations. Analyses of self-reported vaccinations excluded participants who received a vaccine during pregnancy, but were unsure if it was influenza. Statistical significance was evaluated through the Wald Chi-square test. Variables found to be significant at *P*<0.20 from bivariate analyses were included in manual forward step-wise multivariable logistic regression models to evaluate associations with influenza vaccination. Variables with the smallest *P-*value from bivariate analyses were added one at a time to the forward step-wise regression models and removed at a *P<*0.20 significance level. Values of *P*<0.05 were considered statistically significant. We used tolerance values to assess collinearity among independent variables and Hosmer-Lemeshow to assess goodness-of-fit of the final adjusted model. We report unadjusted and adjusted odds ratios, and 95% CIs. We used SAS V.9.4 (SAS Institute, Inc., Cary, North Carolina) for all analyses.

## Results

### Sample characteristics

Of 886 eligible postpartum women, 842 agreed to participate. We surveyed 749 postpartum women in 15 SESAL healthcare facilities and 93 in two IHSS facilities. The most prevalent departments of residence were Cortés (26.6%), Francisco Morazán (25.4%), and Olancho (11.8%) (Table 1). Of all participants, 80.1% were 18–34 years of age, 87.3% were Mestiza (mixed Amerindian and European ancestry), 74.1% were housewives, 46.9% had ≤primary school education, 62.1% had at least one other child, and 4.8% had a concurrent chronic disease. Additionally, 10.0% had a premature delivery, 98.2% received prenatal care by a healthcare professional, and 80.6% were recommended the vaccine by a healthcare worker during prenatal check-ups.

**Table 1:** Demographics, prenatal care, and influenza vaccination coverage of 842 postpartum women, Honduras, August 20 to October 8, 2018.

Characteristic	n (%)
Age (in years)	
<18	65 (7.7)
18–34	674 (80.1)
>34	103 (12.2)
Department of residence	
Cortés	224 (26.6)
Francisco Morazán	214 (25.4)
Olancho	99 (11.8)
Colón	66 (7.8)
Comayagua	59 (7.0)
Other	180 (21.4)
Race	
White	12 (1.4)
Mestiza	735 (87.3)
Mulatta	6 (0.7)
Other	14 (1.7)
Do not know	75 (8.9)
Educational attainment	
No formal education/primary school	395 (46.9)
Middle school	239 (28.4)
High school	154 (18.3)
University	54 (6.4)
Occupation	
Salaried employee	118 (14.0)
Self-employed	44 (5.2)
Unemployed	14 (1.7)
Housewife	624 (74.1)
Student	42 (5.0)
Marital status	
Single	74 (8.8)
Married	148 (17.6)
Cohabitation	608 (72.2)
Other	12 (1.4)
Concurrent chronic disease^a^	40 (4.8)
Gestational age (n = 821)	
<37 weeks	82 (10.0)
≥37 weeks	739 (90.0)
Number of other children in household	
≥3	150 (17.8)
2	158 (18.8)
1	215 (25.5)
0	319 (37.9)
Vaccination status of other children in household (n = 815)	
Vaccinated	285 (35.0)
Not vaccinated	211 (25.9)
No other children	319 (39.1)
Number of prenatal visits (n = 825)	
>8	171 (20.7)
6–8	355 (43.0)
<6	299 (36.3)
Receipt of vaccination recommendation by a healthcare worker during prenatal check-up	679 (80.6)
Health system	
Ministry of Health of Honduras	749 (89.0)
Honduran Social Security Institute	93 (11.0)
Medical setting	
Hospital	807 (95.8)
Clinic	35 (4.2)
Distance to nearest vaccination site (in kilometers)	
≤1	198 (23.5)
>1–5	350 (41.6)
>5–10	183 (21.7)
>10	111 (13.2)
Vaccinated for seasonal influenza (self-reported)^b^ (n = 797)^c^	656 (82.3)
Vaccinated for seasonal influenza (verified)^b^ (n = 606)^d^	465 (76.7)
Vaccinated for seasonal influenza (verified)^b^, pregnancy ≥37 weeks (n = 534)	417 (78.1)

n = 842 unless indicated otherwise due to non-response

^a^ 27 had asthma, 8 had diabetes mellitus, 5 had chronic heart disease, 1 had chronic kidney disease.

^b^ Participants self-reported their vaccination status, which we subsequently verified with vaccination cards or medical records from the Latin American Perinatology Center.

^c^ Excluded 45 participants who received a vaccine during pregnancy, but were unsure if it was influenza.

^d^ Excluded 236 participants with unverified influenza vaccinations.

### Influenza vaccination

Of all 842 participants, 797 self-reported their vaccination status for seasonal influenza, of whom 656 (82.3%; 95% CI: 79.7–85.0%) professed vaccination (Table 1). Forty-five participants were excluded who received a vaccine during pregnancy, but were unsure if it was influenza. Of all participants, we were able to verify 606 records via vaccination cards or medical records, of whom 465 (76.7%; 95% CI: 73.3–80.1%) were vaccinated. Two hundred thirty-six participants with unverified vaccinations were excluded. Of the 606 participants with verified records, 534 had term pregnancy, of whom 417 (78.1%) were vaccinated.

The final model for verified influenza vaccination included education, concurrent chronic disease, vaccination status of other children, and received vaccination recommendation from a healthcare worker (Table 2). Adjusting for the other variables in the model, the odds of verified influenza vaccination were 16.46 times higher for postpartum women who received vaccination recommendations from healthcare workers during prenatal check-up (95% CI: 9.73–27.85), 5.00 times higher for those with a concurrent chronic disease (95% CI: 1.25–20.07), and 2.28 times higher for those who had other children vaccinated for influenza in the household (95% CI: 1.19–4.39). The Hosmer-Lemeshow goodness-of-fit test demonstrated the model fit was adequate (p = 0.62). Tolerance values were >0.95, so there was no evidence of collinearity. Results were similar when including self-reported vaccinations, except that concurrent chronic disease was not significant in the final model (S1 Table).

**Table 2:** Associations between demographics and prenatal care, and influenza vaccination (verified^a^), postpartum women (n = 563)^b^, Honduras, August 20 to October 8, 2018.

Variable	OR (95% CI)	*P-*value	aOR^c^ (95% CI)	*P-*value
Age group (Ref: <18 years)		0.369		
18–34 years	1.40 (0.71–2.77)		–	
≥35 years	1.90 (0.78–4.65)		–	
Department of residence (ref: Cortés)		0.014		
Olancho	0.91 (0.48–1.73)		–	
Francisco Morazán	2.35 (1.36–4.07)		–	
Colón	1.89 (0.74–4.84)		–	
Comayagua	2.85 (1.06–7.70)		–	
Other	1.92 (1.06–3.47)		–	
Educational attainment (Ref: no formal education/primary school)		0.084		0.147
Middle school	0.58 (0.36–0.94)		0.62 (0.34–1.11)	
High school	0.67 (0.38–1.16)		0.99 (0.50–1.96)	
University	0.48 (0.22–1.01)		0.43 (0.17–1.05)	
Occupation (Ref: housewife)		0.067		
Salaried employee	0.63 (0.37–1.08)		–	
Self-employed	0.58 (0.26–1.32)		–	
Unemployed	0.30 (0.09–1.00)		–	
Student	1.91 (0.56–6.52)		–	
Marital status (Ref: married)		0.240		
Single	0.52 (0.24–1.10)		–	
Accompanied	0.94 (0.54–1.61)		–	
Other	1.75 (0.20–15.05)		–	
Concurrent chronic disease (Ref: no)	2.93 (0.88–9.75)	0.081	5.00 (1.25–20.07)	0.023
≥37 weeks gestational age (Ref: <37 weeks)	1.22 (0.64–2.31)	0.548	–	
Number of other children in household (Ref: 0)		0.527		
≥3	1.60 (0.84–3.00)		–	
2	1.22 (0.69–2.15)		–	
1	1.18 (0.72–1.94)		–	
Vaccination status of other children in household (Ref: not vaccinated)		<0.001		0.047
Vaccinated	3.03 (1.73–5.29)		2.28 (1.19–4.39)	
No other children in household	1.31 (0.83–2.08)		1.36 (0.77–2.41)	
Number of prenatal visits (Ref: <6)		0.155		
>8	1.75 (0.99–3.08)		–	
6–8	1.21 (0.77–1.90)		–	
Received vaccination recommendation by a healthcare worker during prenatal check-up (Ref: no)	16.30 (9.89–26.89)	<0.001	16.46 (9.73–27.85)	<0.001
Ministry of Health of Honduras (Ref: Honduran Social Security Institute)	1.39 (0.80–2.42)	0.249	–	
Gave birth in clinic (Ref: hospital)	2.18 (0.75–6.30)	0.152	–	
Distance to nearest vaccination site (in kilometers) (Ref: >10)		0.681		
≤1	0.90 (0.45–1.82)		–	
>1–5	1.00 (0.52–1.91)		–	
>5–10	1.30 (0.63–2.71)		–	

Ref: reference; OR: odds ratio; aOR: adjusted odds ratio; CI: confidence interval

^a^ Verified with vaccination cards and medical records from the Latin American Perinatology Center.

^b^ Analyses excluded participants with unverified influenza vaccinations and those who did not provide complete demographics.

^c^ Adjusted for the other variables listed in the model.

### Knowledge of influenza vaccination

Almost all participants knew that pregnant women have a high risk of influenza complications (97.1%; 95% CI: 96.0–98.3%) and vaccination protects against those complications (96.5%; 95% CI: 95.2–97.8%) (Table 3). However, 17.9% and 28.0% of the participants were unware that influenza may be transmitted from contact with asymptomatic cases (95% CI: 15.1–20.8%) and infected birds or pigs (95% CI: 24.2–31.8%), respectively.

**Table 3:** Knowledge of influenza vaccination, postpartum women, Honduras, August 20 to October 8, 2018.

	Total participants^a^	Agreed	
Knowledge	N	n	% (95% CI)
Influenza causes serious illness	777	749	96.4 (95.1–97.7)
Pregnant women have a higher risk of complications from influenza	807	784	97.1 (96.0–98.3)
Influenza may be transmitted from person to person	760	695	91.4 (89.5–93.4)
Influenza may be transmitted even if the infected person feels well	691	567	82.1 (79.2–84.9)
Influenza may be transmitted if people touch their mouths or noses with contaminated hands	781	725	92.8 (91.0–94.6)
Influenza may be transmitted from birds or pigs to people	543	391	72.0 (68.2–75.8)
People may contract influenza even if they have contracted influenza before	686	599	87.3 (84.8–89.8)
Aware of an influenza vaccine	802	797	99.4 (98.8–99.9)
The influenza vaccine protects against influenza complications	776	749	96.5 (95.2–97.8)
The influenza vaccine is safe for mothers and their infants	786	753	95.8 (94.4–97.2)

CI: confidence interval

^a^ Excluded postpartum women who did not respond.

### Attitudes regarding influenza vaccination

Of 465 participants who were vaccinated for seasonal influenza, 424 perceived some benefits from this vaccine (91.2%; 95% CI: 88.6–93.8%), 267 cited easy access to vaccines (e.g., offered the vaccine during prenatal check-up appointments) (57.3%; 95% CI: 52.9–61.9%), and 160 cited healthcare provider recommendations (34.4%; 95% CI: 30.1–38.7%) as reasons for vaccination (Table 4).

**Table 4:** Reasons for receiving influenza vaccination, postpartum women (n = 465), Honduras, August 20 to October 8, 2018.

Reasons	Total agreed	Agreed % (95% CI)
*Easy access*	267	57.3 (52.9–61.9)
Offered vaccine during prenatal check-up appointment	258	55.5 (51.0–60.0)
Favorable vaccination schedules	57	12.3 (9.3–15.2)
Obtained permission from workplace for vaccination	28	6.0 (3.9–8.2)
*Perceived benefits*	424	91.2 (88.6–93.8)
Perceived vaccines as beneficial for mother and infant	395	84.9 (81.7–88.2)
Believed vaccine can protect against serious influenza	253	54.4 (49.9–59.0)
Perceived personal risk for influenza	199	42.8 (38.3–47.3)
Perceived vaccine side effects as less harmful than influenza	170	36.6 (32.2–41.0)
Preferred vaccination over spending money on treatment	161	34.6 (30.3–39.0)
*Previous experiences*	112	24.1 (20.2–28.0)
No problems with previous vaccination	39	8.4 (5.9–10.9)
Have not observed negative effects of vaccination	96	20.6 (17.0–24.3)
*Peer influence*	63	13.5 (10.4–16.7)
Urged to get vaccinated by family members	46	9.9 (7.2–12.6)
Urged to get vaccinated by other pregnant women	29	6.2 (4.0–8.4)
Observed other pregnant women getting vaccinated	28	6.0 (3.9–8.2)
*Health establishment counseling*	160	34.4 (30.1–38.7)
Urged to get vaccinated by a doctor or nurse	125	26.9 (22.8–30.9)
Listened to promotional outreach on vaccinations at a healthcare facility	93	20.0 (16.4–23.6)
*Aware of vaccine benefits from mass media*	98	21.1 (17.4–24.8)

CI: confidence interval

Composite subheadings (e.g., easy access) included at least one positive response for one of the listed reasons

Of 141 unvaccinated participants, 66 cited difficulties accessing the vaccine (e.g., vaccine was not offered by healthcare workers or was unavailable on-site) (46.8%; 95% CI: 38.5–55.1%), 64 declined the vaccine primarily due to fear of adverse effects (45.4%; 95% CI: 37.1–53.7%), and 32 were not advised to get vaccinated (22.7%; 95% CI: 15.7–29.7%) (Table 5).

**Table 5:** Reasons for not receiving influenza vaccination, postpartum women (n = 141), Honduras, August 20 to October 8, 2018.

Reasons	Total agreed	Agreed % (95% CI)
*Limited access*	66	46.8 (38.5–55.1)
Vaccine was not offered	47	33.3 (25.5–41.2)
Vaccine was not available in healthcare facility	13	9.2 (4.4–14.1)
Did not have time to get vaccinated	7	5.0 (1.3–8.6)
Did not know where to go for vaccine	5	3.5 (0.5–6.6)
Vaccination center was too far away	3	2.1 (0–4.5)
Vaccination center located in dangerous area	2	1.4 (0–3.4)
Inconvenient hours for vaccination	1	0.7 (0–2.1)
*Rejection*	64	45.4 (37.1–53.7)
Fear of side effects	30	21.3 (14.4–28.1)
Fear of harm to infant	23	16.3 (10.1–22.5)
Fear of needles or pain caused by injection	20	14.2 (8.4–20.0)
Fear of contracting influenza	19	13.5 (7.8–19.2)
Believed vaccine is ineffective	15	10.6 (5.5–15.8)
Pressure from friends and family to not get vaccinated	11	7.8 (3.3–12.3)
Believed influenza does not cause serious illness	7	5.0 (1.3–8.6)
*Not advised to be vaccinated*	32	22.7 (15.7–29.7)

CI: confidence interval

Composite subheadings (e.g., limited access) included at least one positive response for one of the listed reasons

## Clinical manifestations of vaccination

Of 465 postpartum women who were vaccinated for influenza, 101 (21.7%; 95% CI: 18.0–25.5%) reported mild or moderate untoward reactions after vaccination, including vaccination site pain, general discomfort, flu-like symptoms, and fever (Table 6).

**Table 6:** Clinical manifestations seven days after vaccination, postpartum women (n = 465), Honduras, August 20 to October 8, 2018.

Clinical manifestation	n	% (95% CI)
Pain at the vaccination site	58	12.5 (9.5–15.5)
General discomfort	18	3.9 (2.1–5.6)
Flu-like symptoms	15	3.2 (1.6–4.8)
Fever	14	3.0 (1.5–4.5)
Inflammation at the vaccination site	8	1.7 (0.5–2.9)
Headache	4	0.9 (0.2–1.7)
Urticaria	3	0.6 (0–1.4)
Hematoma at the vaccination site	2	0.4 (0–1.0)
Dizziness	1	0.4 (0–0.6)

CI: confidence interval

## Discussion

Over three-quarters of postpartum women with verified vaccinations and term pregnancy were vaccinated for influenza. Although lower than SESAL’s goal of 95%, which was proposed to exceed the influenza vaccination herd immunity threshold established for high risk groups in the United States [37, 38], this coverage was similar to that reported by PAHO for pregnant women in Honduras (78%), Costa Rica (72%), and El Salvador (82%) in 2017 [17]. Seasonal influenza vaccination coverage rates for pregnant women in Central America are among the highest worldwide [18, 39]. Differences between Central American countries may be attributed to different vaccination campaigns, communication activities, surveillance systems, medical practices, funding schemes, attitudes towards vaccination, and previous experiences with influenza.

High seasonal influenza vaccination coverage among postpartum women in Honduras may be attributed in part to the Vaccine Law of the Republic of Honduras (implemented in 2014), which mandates that all residents, including pregnant women, be vaccinated for all vaccine-preventable diseases determined by SESAL, which includes seasonal influenza [40]. Seasonal influenza vaccines are available free-of-charge during prenatal care and in public health centers and other IHSS healthcare facilities countrywide [41]. High coverage may also be credited to SESAL’s Expanded Program of Immunization (EPI), which is responsible for implementation of vaccination campaigns of high risk groups [42]. In 2015 and 2016, EPI received awards from the Global Vaccine and Immunization Alliance (GAVI) for high coverage and effective vaccine management [40]. Pregnant women were also identified as high risk groups for 2009 pandemic H1N1 and action was taken to protect this particular group through vaccination [43].

Most participants believed the influenza vaccine protected them from influenza and was safe for them and their children. This study was conducted three months after the launch of influenza vaccination campaigns, which includes educational components that likely increased participants’ knowledge about vaccination benefits. However, the main knowledge gaps concerned virus’s ability to be transmitted from animals to humans and from asymptomatic patients. Educational programs tailored at increasing knowledge of influenza’s transmissibility may be beneficial, particularly in locations where animal-human contacts are likely to occur. Considering 11.8% of participants in this study were <18 years of age, it may be prudent to include educational materials in the classroom.

Similar to other studies of pregnant women [18, 19], key incentives for vaccination for seasonal influenza included easy access to vaccines, healthcare worker recommendations, and perceived benefits of vaccination. The finding that vaccine recommendations by healthcare personnel had the strongest associations with influenza vaccination is in accord with other studies [18, 19, 44, 45], highlighting the importance of obstetric healthcare personnel directly recommending and offering vaccinations to pregnant women during prenatal check-up appointments. Concerns expressed by women regarding the safety of influenza vaccinations may be mitigated when recommendations are provided directly from their healthcare providers [46]. Patient counseling conveys that influenza vaccines may reduce risk of respiratory illnesses and hospital admissions for patients and their infants <6 months of age.

The finding that participants who had other children vaccinated for seasonal influenza were more likely to be vaccinated than those without other vaccinated children is consistent with a study of older adults in Germany [47]. This finding may suggest that when pregnant women bring their children to a healthcare facility to be vaccinated, those women should be offered the influenza vaccine as well. Prevention of influenza among other children and family members in close contact with pregnant women also decreases the risk of infection in the mother.

Our finding that postpartum women with comorbidities were more likely to be vaccinated for seasonal influenza is in accord with some studies of pregnant women [48, 49], whereas others did not find an association [44, 50–52]. Pregnant women with concurrent chronic diseases are over three times more likely to be hospitalized during all three trimesters for respiratory illness following influenza infection [53, 54]. Both pregnant women and individuals with underlying comorbidities are target groups for vaccination in Honduras [25]. It is conceivable that pregnant women with chronic diseases interact more regularly with physicians in the healthcare system, and may therefore be more likely to receive a vaccination recommendation and/or be offered vaccination.

Although other studies demonstrated associations between seasonal influenza vaccination and demographics (unemployment, married, maternal age, socioeconomic status, education, number of pregnancies, and number of prenatal visits) [45, 55–57], these factors were not associated with influenza vaccination in this study.

The most frequently cited barrier to seasonal influenza vaccination was inaccessibility to vaccine, which is supported by other research [9, 46]. Specifically, a third of unvaccinated patients mentioned they were not offered the vaccine or informed from workers at the healthcare facility that an influenza vaccine was available. Healthcare workers may not offer the influenza vaccine due to their own lack of knowledge regarding vaccine safety and efficacy or fear of consequences of liability given adverse side effects [56]. Furthermore, there may be lack of clarity regarding whether primary care physicians or obstetrician-gynecologist specialists are responsible for vaccine recommendations and administration [58]. On-site provision of influenza vaccines in prenatal clinics and ensuring obstetric healthcare providers are informed about the national technical guidelines regarding influenza vaccinations for pregnant women may increase vaccination coverage [19].

The finding that fear of side effects, needles or pain caused by injection, and contracting influenza were common reasons for non-vaccination is consistent with other studies [9, 18, 19, 44, 57]. Furthermore, 27 vaccinated participants reported fever or flu-like symptoms within one week after vaccination. Educational campaigns may help dispel misconceptions regarding safety and efficacy of vaccination during pregnancy. Educational activities should not be limited to immediately before vaccination campaigns, but need to be done throughout the year including times of low virus circulation.

This study had several limitations. First, we included women recruited from hospitals and clinics from SESAL and IHSS, which may not be representative of all postpartum women in Honduras. Second, this was a cross-sectional study, so we were unable to make causal inferences about relationships between KAP regarding influenza and vaccination. Third, our logistic regression analyses resulted in odds ratios with wide confidence intervals, which indicates high variability and should be interpreted with caution. Fourth, there may have been social desirability bias in self-reported responses regarding vaccination knowledge and behavior. Fifth, there may have been recall bias if vaccinated patients recalled certain factors (e.g., provider recommendations) differently than unvaccinated patients. Sixth, there may have been response bias if vaccinated patients who had greater knowledge of influenza vaccination were more likely to participate than unvaccinated patients. Seventh, we did not collect data on participants who refused to participate. Finally, this study did not include assessment of participant preferences for where they wanted to receive information about influenza vaccination.

Notwithstanding these limitations, our study included a large sample of postpartum women in hospitals and clinics in Honduras. Second, we were able to verify most of the influenza vaccinations with vaccination cards and medical records. Third, we had a 95% response rate. Fourth, we are unaware of any other KAP studies of seasonal influenza vaccination among pregnant or postpartum women in Honduras.

Pregnant women and their infants benefit from maternal influenza vaccination. Influenza vaccinations were well received among postpartum women in Honduras, which may be attributed to its robust vaccination program. Our results suggest that increasing vaccination recommendations from clinicians may increase seasonal influenza vaccination rates. Vaccine advice should be included in routine prenatal care and provided consistently throughout vaccination campaigns.

## Supporting information

S1 Table Associations between demographics and prenatal care, and influenza vaccination (self-reported), postpartum women (n = 738), Honduras, August 20 to October 8, 2018. (DOCX)

S1 Survey (DOCX)

S1 File Equation used to obtain sample sizes for survey of postpartum women. (DOCX)
