# Supplementary material for: Knowledge, attitudes, and practices of seasonal influenza vaccination in postpartum women, Honduras
Source: PLoS One. 2021 Feb 11;16(2):e0246385. doi: 10.1371/journal.pone.0246385 (PMC7877664; doi:10.1371/journal.pone.0246385)
Supplement: S1 Table — (DOCX) [file pone.0246385.s001.docx]

| **S1 Table. Associations between demographics and prenatal care, and influenza vaccination (self-reported), postpartum women (n = 738)^a^, Honduras, August 20 to October 8, 2018** | | | | |
| --- | --- | --- | --- | --- |
| Variable | OR (95% CI) | *P-*value | aOR^b^ (95% CI) | *P-*value |
| Age group (Ref: <18 years) |  | 0.260 |  |  |
| 18-34 years | 1.61 (0.83–3.12) |  | – |  |
| ≥35 years | 2.01 (0.84–4.80) |  | – |  |
| Department of residence (ref: Cortés) |  | 0.001 |  |  |
| Olancho | 1.36 (0.74–2.51) |  | – |  |
| Francisco Morazán | 2.81 (1.64–4.81) |  | – |  |
| Colón | 2.53 (1.02–6.29) |  | – |  |
| Comayagua | 3.61 (1.34–9.55) |  | – |  |
| Other | 2.51 (1.41–4.46) |  | – |  |
| Educational attainment (Ref: no formal education/primary school) |  | 0.049 |  |  |
| Middle school | 0.57 (0.36–0.91) |  | – |  |
| High school | 0.61 (0.36–1.04) |  | – |  |
| University | 0.48 (0.23–0.98) |  | – |  |
| Occupation (Ref: housewife) |  | 0.067 |  |  |
| Salaried employee | 0.66 (0.39–1.09) |  | – |  |
| Self-employed | 0.62 (0.29–1.36) |  | – |  |
| Unemployed | 0.33 (0.11–1.00) |  | – |  |
| Student | 2.05 (0.61–6.82) |  | – |  |
| Marital status (Ref: married) |  | 0.120 |  |  |
| Single | 0.45 (0.22–0.94) |  | – |  |
| Accompanied | 0.89 (0.53–1.51) |  | – |  |
| Other | 1.54 (0.19–12.81) |  | – |  |
| Concurrent chronic disease (Ref: no) | 2.27 (0.68–7.52) | 0.180 | 3.70 (0.97–14.10) | 0.056 |
| ≥37 weeks gestational age (Ref: <37 weeks) | 1.26 (0.68–2.34) | 0.469 | – |  |
| Number of other children in household (Ref: 0) |  | 0.356 |  |  |
| ≥3 | 1.71 (0.93–3.16) |  | – |  |
| 2 | 1.30 (0.75–2.25) |  | – |  |
| 1 | 1.11 (0.68–1.79) |  | – |  |
| Vaccination status of other children in household (Ref: not vaccinated) |  | <0.001 |  | 0.007 |
| Vaccinated | 3.12 (1.81–5.38) |  | 2.60 (1.40–4.81) |  |
| No other children in household | 1.32 (0.85–2.06) |  | 1.25 (0.74–2.10) |  |
| Number of prenatal visits (Ref: <6) |  | 0.473 |  |  |
| >8 | 1.40 (0.81–2.43) |  | – |  |
| 6-8 | 1.06 (0.69–1.64) |  | – |  |
| Received vaccination recommendation by a healthcare worker during prenatal check-up (Ref: no) | 15.10 (9.55–23.88) | <0.001 | 14.69 (9.19–23.48) | <0.001 |
| Ministry of Health of Honduras (Ref: Honduran Social Security Institute) | 1.59 (0.92–2.73) | 0.094 | – |  |
| Gave birth in clinic (Ref: hospital) | 1.53 (0.53–4.41) | 0.436 | – |  |
| Distance to nearest vaccination site (in kilometers) (Ref: >10) |  | 0.891 |  |  |
| ≤1 | 0.88 (0.45–1.73) |  | – |  |
| >1–5 | 0.89 (0.48–1.67) |  | – |  |
| >5–10 | 1.08 (0.53–2.18) |  | – |  |
| Ref: reference; OR: odds ratio; aOR: adjusted odds ratio; CI: confidence interval; | | | | |
| ^a^ Excluded participants who received a vaccine during pregnancy but were unsure if it was influenza, and did not provide complete demographics. | | | | |
| ^b^ Adjusted for the other variables listed in the model. | | | | |
