# Supplementary material for: Knowledge, attitudes, and practices of seasonal influenza vaccination in postpartum women, Honduras
Source: PLoS One. 2021 Feb 11;16(2):e0246385. doi: 10.1371/journal.pone.0246385 (PMC7877664; doi:10.1371/journal.pone.0246385)
Supplement: S1 Survey — (DOCX) [file pone.0246385.s002.docx]

**Encuesta a mujeres postparto**

[*Nota: los textos en color* ***celeste*** *estarán ocultos para los encuestados*]

[*Nota: este formulario no se utilizará impreso, solo en tableta*]

***Datos demográficos de la participante***

1. Nombre del establecimiento: ____________________________________
2. Fecha de la entrevista: ____/_____/_____ (dd/mm/aaaa)
3. Código para el estudio: _____________________________________________
4. Nombre de la persona entrevistada: ____________________________________
5. ¿Cuántos años cumplidos tiene? ________ años.
6. ¿Cuál es su fecha de nacimiento? ____/_____/_____ (dd/mm/aaaa)
7. Departamento donde reside: _________________
8. Municipio donde reside: ___________________
9. ¿Hasta qué nivel de estudios ha llegado usted?:
10. No sabe leer ni escribir, y no ha realizado estudios formales
11. Sabe leer y escribir, pero no ha realizado estudios formales
12. Primaria incompleta
13. Primaria completa
14. Secundaria incompleta
15. Secundaria completa
16. Bachillerato o diversificado incompleto
17. Bachillerato o diversificado completo
18. Estudios universitarios
19. Maestría profesional, especialización o postgrado
20. Doctorado
21. No responde
22. ¿Cuál es su empleo?:
23. Empleada con salario

a.1.  Empleado de salud

1. Trabajando por cuenta propia
2. Sin trabajo pro más de 1 año
3. Sin trabajo por menos de 1 año
4. Ama de casa
5. Estudiante
6. No puede trabajar
7. Otro. ¿Cuál? ___________________________________________.
8. ¿Con cuál de las siguientes etnias usted se identifica?
9. Blanca;
10. Mestiza;
11. Mulata;
12. Descendiente africana;
13. Indígena

Especificar:

Lenca,

Garífuna,

Tolupanes,

Misquitos,

Pech,

Chortís,

Tawahka,

Otra categoría indígena ¿Cuál? __________)

1. Otra etnia ¿Cuál? __________
2. No sabe,
3. No responde.
4. ¿Cuál es su estado actual civil? [leer opciones en voz alta]
5. Soltera (nunca casada o acompañada)
6. Casada
7. Acompañada o en unión libre
8. Divorciada
9. Separada
10. Viuda
11. No responde.

***Pregunta sobre el embarazo actual***

1. ¿Cuál es la fecha de su última regla? ____/_____/_____ dd / mm / aaaa

No sabe / No recuerda

***Preguntas sobre vacunación a los otros hijos***

1. Además del que acaba de nacer ¿Tiene usted más hijos?

Sí  No  No responde

*(Si la respuesta es No, o No responde, pase a la pregunta 17)*

1. ¿Cuántos hijos tiene Ud? _______ hijos
   1. ¿Qué edad tiene su hijo #1? _____  años meses
   2. ¿Qué edad tiene su hijo #2? _____  años  meses
   3. ¿Qué edad tiene su hijo #3? _____  años  meses
2. ¿Sus hijos han sido alguna vez vacunados contra la influenza?

Sí;  No;  No sabe  No responde;  No aplica (primer hijo, o único hijo vivo).

**En la siguiente sección le leeremos una serie de aseveraciones sobre la influenza y la vacunación contra la misma. Le solicitamos que nos diga si está de acuerdo o en desacuerdo con cada una. También puede decirnos que no sabe, o que no desea comentar al respecto.**

***Percepción de la severidad de la enfermedad***

1. La influenza o gripe puede causar una enfermedad grave.

De acuerdo;  En desacuerdo;  No sabe  No responde

1. Todas las personas están en riesgo de adquirir la influenza, pero las mujeres embarazadas tienen mayor riesgo de tener complicaciones severas que resulten en hospitalizaciones o muerte.

De acuerdo;  En desacuerdo;  No sabe  No responde

***Conocimientos sobre la transmisión de la influenza***

1. La influenza o gripe puede transmitirse entre las personas

De acuerdo;  En desacuerdo;  No sabe  No responde

1. Aunque se sientan bien, una persona pueden transmitir el virus de la influenza a otra persona.

De acuerdo;  En desacuerdo;  No sabe  No responde

1. Las personas pueden adquirir la influenza si toca su boca o nariz después de haber tocado algo contaminado con el virus de la influenza.

De acuerdo;  En desacuerdo;  No sabe  No responde

1. La influenza puede ser transmitida de las aves o los cerdos a las personas

De acuerdo;  En desacuerdo;  No sabe  No responde

1. Una persona puede adquirir la influenza aunque se haya enfermado antes de influenza.

De acuerdo;  En desacuerdo;  No sabe  No responde

***Percepción de la efectividad de la vacuna contra la influenza***

1. Existe una vacuna para prevenir la influenza o gripe

De acuerdo;  En desacuerdo;  No sabe  No responde

1. La vacuna contra influenza o gripe puede proteger a una persona contra la influenza y las complicaciones que esta pueda traer.

De acuerdo;  En desacuerdo;  No sabe  No responde

***Percepción de la seguridad de la vacuna contra la influenza***

1. La administración de la vacuna contra la influenza durante el embarazo es segura tanto para usted como para su hijo.

De acuerdo;  En desacuerdo;  No sabe  No responde

**Ahora le voy a hacer unas preguntas sobre su control prenatal**

***Preguntas sobre factores que favorecen la vacunación***

1. ¿Asistió a la clínica para su control prenatal por su reciente embarazo?

Sí

No (Pase a la pregunta 31)

No sabe  No responde

1. ¿Cuántas veces visitó la clínica para recibir su control prenatal? _______ veces.

No sabe  No responde

Mover a otra sección y colocar un subtítulo (colocar fecha para ver si estaba disponible)

**Ahora le haré unas preguntas sobre la vacuna contra la influenza estacional**

***Acceso facilitado a la vacunación contra la influenza***

1. ¿El médico en la enfermera que le atendió durante el control prenatal en el reciente embarazo le recomendó que se vacunara contra la influenza?

Sí  No  No sabe  No responde

1. ¿Dónde la refirieron para que fuera vacunada contra la influenza estacional?

Al establecimiento de salud del control prenatal, durante una cita de control prenatal

Al establecimiento de salud del control prenatal pero en otra cita diferente a la del control prenatal

A otro establecimiento de salud de la Secretaría de Salud donde estaba disponible la vacuna de influenza?

A un proveedor privado especializado en vacunas

No la refirieron. Dejaron que usted decidiera dónde vacunarse.

***Vacunación contra la influenza***

1. ¿Recibió la vacuna contra la influenza estacional durante el embarazo que finalizó en el 2018?

Sí

Recibió una vacuna durante el embarazo, pero no sabe si era la de influenza

No *(Pase a la pregunta 40)*

No responde

Si es Sí:

1. ¿Cuáles fueron los motivos por el que decidió vacunarse contra la influenza durante el reciente embarazo? (*dejar que el participante exprese sus motivos, y después leerle el listado de opciones*)

***Fácil acceso a la vacuna***

Me ofrecieron la vacuna durante mi cita de control prenatal

Los horarios de vacunación me son favorables

Me dieron permiso en mi trabajo para ir a vacunarme

***Percepción del beneficio propio***

Las vacunas son buenas para mí y mi bebé

Considero que la vacuna puede protegerme de influenza grave

Considero que soy una persona en riesgo de enfermar por influenza

***Percepción del beneficio sobre el riesgo***

Considero que los efectos adversos de la vacuna contra la influenza estacional no son tan malos ni para mí, ni para mi hijo, en comparación con desarrollar la influenza

Prefiero ponerme la vacuna que gastar en el tratamiento por la enfermedad (influenza)

***Experiencias previas***

Antes me vacuné contra la influenza y no me pasó nada malo

No he visto que le haya sucedido algo malo a algún conocido por vacunarse contra la influenza

***Influencia de familiares/amigos/compañeros de trabajo***

He visto que otras mujeres embarazadas se vacunan contra la influenza

Mis familiares me pidieron que me vacunara contra la influenza

Otras amigas embarazadas me recomendaron que me vacunara contra la influenza

***Consejería por el establecimiento de salud***

Un médico o una enfermera me recomendaron que me vacunara contra la influenza

Nos dieron una charla en un establecimiento de salud dónde se nos aconsejaba que nos vacunáramos contra la influenza.

***Influencia de los medios de comunicación***

Me enteré (en la radio, televisión, periódico, internet u otro medio masivo de comunicación) que era bueno que me vacunara contra la influenza

Otro motivo. ¿Cuál?_________________________________________

1. ¿Presentó algún síntoma en los 7 días posteriores a la vacunación del 2018?

Sí  No  No recuerda  No responde

Si es Sí:

1. ¿Cuál síntoma presentó?

Malestar general;

Dolor en el sitio de la vacunación;

Hinchazón y/o enrojecimiento en el sitio de vacunación

Hematoma en el sitio de vacunación

Urticaria

Reacción alérgica

Fiebre o sensación de fiebre;

Mareos

Síntomas similares a una gripe;

Otro. ¿Cuál? ___________________

No sabe  No responde

***Verificación de la vacunación***

1. Para este estudio, necesitamos verificar la fecha de vacunación. ¿Tiene usted el carné de vacunación aquí?

Sí;  No;  No sabe  No responde

Si es No

1. ¿Puedo llamarle por teléfono en pocos días para que usted me lea las fechas de vacunación de su carné? *(Esta pregunta aplica tanto a las que dijeron que sí se vacunaron, como a las que recibieron una vacuna durante el embarazo, pero no sabían si era la de influenza)*.

Sí;  No;

Si es Sí:

- 1. ¿Cuál es su número de teléfono? _____________
  2. ¿Cuándo debo llamarle? Fecha: ___/___/_____, hora: _____

Si es No:

1. ¿Recuerda en cuál centro de salud fue vacunada contra la influenza?

Sí;  No;

Si es sí:

- 1. Fecha aproximada en la que fue vacunada: ___/___/_____
  2. Nombre del establecimiento de salud: __________________________________
  3. Ubicación del establecimiento de salud: _________________________________

1. Fecha de vacunación contra la influenza (fecha verificada): _____/_____/______ (dd/mm/aaaa)
2. Fuente de información:

Carné de vacunación  Expediente médico  Llamada telefónica  Visita centro de vacunación

No se logró verificar la fecha de la vacunación.

1. ¿Cuáles fueron los motivos por los que no se vacunó contra la influenza estacional en el 2018?

***Rechazo por temor a efectos adversos***

Tiene temor a enfermar de gripe si se vacuna contra la influenza estacional

Tiene temor de los efectos secundarios para ella

Le da miedo que la vacuna le haga daño a su bebé

***Preocupación por la inyección***

No le gusta vacunarse

Le dan miedo las agujas

Tiene temor al dolor que causa la inyección

***Percepción de falta de utilidad de la vacunación***

No creo que la vacuna realmente evite que yo me enferme

No creo que pueda enfermar gravemente por influenza (no vale la pena vacunarse)

***Limitaciones en el acceso a la vacuna***

Fue al centro de salud a vacunarse, pero no había vacuna disponible

Nadie le dijo que debía vacunarse contra la influenza durante el embarazo

No se le ha ofrecido la vacuna

No sabe a dónde acudir por una vacuna

No tiene tiempo para ir a vacunarse

No le dieron permiso en el trabajo para ir a vacunarse

El centro de vacunación le queda lejos

Es muy peligroso para usted trasladarse al centro de vacunación (debido al crimen)

El centro de vacunación abre en horarios que a usted no le convienen

La vacuna es demasiado cara

***Influencia social de compañeros de trabajo/familiares/amigos***

La vacunación contra la influenza no es aceptada por otras mujeres embarazadas

Sus familiares le dijeron que no se vacunara

Sus amigos le dijeron que no se vacunara

Otro ¿Cuál?_______________________________________________________

1. ¿Qué tan lejos queda de su casa el centro de vacunación? _______kilómetros

**Muchas gracias por haber participado en esta encuesta.**

**Se complementará la encuesta con la información del expediente de la paciente:**

1. ¿En el expediente de la paciente se encuentra el formulario del CLAP?

Sí;  No;  no se logró revisar el expediente.

- 1. Si es Sí, ¿Está anotada la fecha en la cual recibió la vacuna contra la influenza estacional?

Sí;  No.

Si es no, ¿Cuáles fueron los motivos por los que no se tuvo acceso al formulario del CLAP?

No se nos permitió el acceso al expediente

A pesar que nos permitieron el acceso al expediente, la mujer postparto no llevó el carné del CLAP al hospital

Otro motivo ¿Cuál? _____________________________________

1. En el expediente médico de la paciente está anotada algunas de las siguientes enfermedades crónicas:

| **Enfermedad** | **Sí** | **No** | **Sin dato** |  | **Enfermedad** | **Sí** | **No** | **Sin dato** |
| --- | --- | --- | --- | --- | --- | --- | --- | --- |
| Enf. crónica del corazón |  |  |  |  | Diabetes mellitus |  |  |  |
| Asma |  |  |  |  | Enf. renal crónica |  |  |  |
| Bronquitis |  |  |  |  | Inmunosupresión (incluye VIH) |  |  |  |
| EPOC |  |  |  |  | Cáncer |  |  |  |
| Fibrosis quística |  |  |  |  | Enfermedad cerebrovascular |  |  |  |

1. Semanas de edad gestacional al momento del parto: ______ semanas (*calculada por el sistema*).
2. ¿Edad gestacional al momento del parto en el expediente? _________semanas. (*si no hay fecha de última regla*)
3. Edad gestacional por ultrasonido: Edad gestacional al momento del ultrasonido: _______semanas. Fecha del ultrasonido _____/_____/_______ (dd/mm/aaaa).

**Questionnaire in English**

[Note: This form will used only in tablet]

1. Healthcare facility name: ____________________________________
2. Date of interview: ____ / _____ / _____ (dd / mm / yyyy)
3. Code assigned to the participant: _____________________________________________
4. Name of the interviewee: ____________________________________
5. How old are you? ________ years.
6. What is your birth date? ____ / _____ / _____ (dd / mm / yyyy)
7. Department of residence: _________________
8. Municipality where you reside: ___________________
9. Educational attainment:

Cannot read or write, and has no formal studies

Literate, but has no formal studies

incomplete primary

full primary

incomplete secondary

Completed secondary

Diversified secondary education or incomplete

Diversified secondary education or full

university

professional expertise, specialization or postgraduate

Doctorate

no response

1. What is your job?:

Salaried employee

(Q10a)  Employee on health

Self-employed

Unemployed for more than 1 year

Unemployed for less 1 yearr

Housewife

student

Cannot work

Other. (Q10b) What? ___________________________________________.

1. Which of the following ethnic groups you identify?

white;

mestiza;

Mulata;

African descendent;

Indigenous

Specify:

Lenca,

Garifuna,

Tolupanes,

Miskito,

Pech,

Chorti,

Tawahka,

Another Indian category (p11h1) What? __________)

Another ethnic group (p11i) What? __________

Does not know,

No response.

1. What is your current marital status? [Read options aloud]

Single (never married or accompanied)

married

Accompanied

Divorced

Separated

Widowed

No response.

1. What is the date of your last period? ____ / _____ / _____ dd / mm / yyyy
2. Besides the one that has just been born: Do you have more children?

Yes  No  no response

1. How many children do you have? _______ children
   1. How old is your child's # 1? _____ (p15a1 old unit) years months
   2. How old is your child # 2? _____ (p15b1 old unit) years months
   3. How old is your child's No. 3? _____ (p15c1 old unit) years months
2. Do your children have ever been vaccinated against influenza?

Yes;  No;  Does not know  no response; Not applicable (first child, or only living son).

**In the next section we will read a series of statements about influenza and vaccination against it. We ask that you tell us whetif you are agree or disagree with each. You can also tell us that you do not know, or do not want to response.**

1. Influenza or flu can cause serious illness.

Agree;  In disagreement;  Does not know  no response

1. Pregnant women have a higher risk of complications from influenza.

Agree;  In disagreement;  Does not know  no response

1. Influenza may be transmitted from person to person

Agree;  In disagreement;  Does not know  no response

1. Influenza may be transmitted even if the infected person feels well.

Agree;  In disagreement;  Does not know  no response

1. Influenza may be transmitted if people touch their mouths or noses with contaminated hands.

Agree;  In disagreement;  Does not know  no response

1. Influenza can be transmitted from birds or pigs to people

Agree;  In disagreement;  Does not know  no response

1. People may contract influenza even if they have contracted influenza before.

Agree;  In disagreement;  Does not know  no response

1. Aware of an influenza vaccine

Agree;  In disagreement;  Does not know  no response

1. The influenza vaccine protects against influenza complications

Agree;  In disagreement;  Does not know  no response

1. The influenza vaccine is safe for mothers and their infants.

Agree;  In disagreement;  Does not know  no response

**Now I'll ask you some questions about your prenatal care**

1. Did you attend the clinic for prenatal care for your recent pregnancy?

Yes  No  Does not know  no response

1. How many times visited the clinic for prenatal care? _______ times.

**Now I'll ask you about the vaccine against seasonal influenza**

1. Did the doctor or nurse who attended you during prenatal care in the recent pregnancy recommended to get vaccinated against influenza?

Yes  No  Does not know  no response

1. Where they referred to it to be vaccinated against seasonal flu?

The same establishment during a prenatal care appointment

The same establishment but other than the appointment of prenatal care

Another health facility of the Ministry of Health where influenza vaccine was available?

A private provider specializing in vaccines

She was refered.Providers let you decide where to get vaccinated.

1. Did you get the seasonal influenza vaccine during pregnancy ending in 2018?

Yes;  Shereceived a vaccination during pregnancy, but do not know if it was the flu

No;  no response

If yes:

1. What were the reasons why you decided to get vaccinated against influenza during the recent pregnancy? (Let the participant express their motives, and then read the list of options)
2. Offered vaccine during prenatal check-up appointment
3. Favorable vaccination schedules
4. Obtained permission from workplace for vaccination
5. Perceived vaccines as beneficial for mother and infant
6. Believed vaccine can protect against serious influenza
7. Perceived personal risk for influenza
8. Perceived vaccine side effects as less harmful than influenza
9. Preferred vaccination over spending money on treatment
10. No problems with previous vaccination
11. Have not observed negative effects of vaccination
12. Observed other pregnant women getting vaccinated
13. Urged to get vaccinated by family members
14. Urged to get vaccinated by other pregnant women
15. Urged to get vaccinated by a doctor or nurse
16. Listened to promotional outreach on vaccinations at a healthcare facility.
17. Aware of vaccine benefits from mass media
18. Another reason. (P32q1) What? ______________________________________
19. Did you file any symptoms within 7 days after vaccination in 2018?

Yes  No  He does not remember  no response

If yes:

1. Which one or more symptoms presented?
2. General discomfort;
3. Pain at the site of vaccination;
4. Inflammation at the site of vaccination
5. Hematoma at the site of vaccination
6. Urticaria
7. Allergic reaction
8. Fever or feeling of fever;
9. Dizziness
10. flu-like symptoms;
11. Other. (P34j1) What? ___________________
12. Does not know
13. no response
14. For this study, we need to verify the date of vaccination. Do you have the vaccination card here?

Yes;  No;  Does not know  no response

It is, is not it

1. Can I phone you in a few days for you to read me the dates of vaccinating your card? (This question applies to those who said yes were vaccinated, as those who received a vaccine during pregnancy but did not know if it was the flu-shot).

Yes;  No;

If yes:

- 1. What is your phone number? _____________
  2. When should I call? Date hour: _____

If No:

1. In which health center were you vaccinated for influenza?

Yes;  No;

If yes:

- 1. Approximate date on which it was vaccinated: ___ / ___ / _____
  2. Healthcare facility name: __________________________________
  3. Location of healthcare facility: _________________________________

1. Date of influenza vaccination (verified date): _____ / _____ / ______ (dd / mm / yyyy)
2. Source of information:
3. Vaccination card
4. medical record
5. Phone call
6. Visit vaccination center
7. It was not possible to verify the date of vaccination.
8. What were the reasons why you was not vaccinated against seasonal influenza during 2018 campaign?
9. Fear of contracting influenza disease
10. Fear of the side effects
11. Fear of harm to infant
12. does not like to be vaccinated
13. Fear of needles
14. Fear of pain caused by injection
15. Believed vaccine is ineffective
16. Believed influenza does not cause serious illness
17. Vaccine was not available in healthcare facility
18. Not advised to be vaccinated
19. Vaccine was not offered
20. Did not know where to go for vaccine
21. Did not have time to get vaccinated
22. Not given time off work to go to get vaccinated
23. Vaccination center was too far away
24. Vaccination center located in dangerous area
25. Inconvenient hours for vaccination
26. The vaccine is too expensive
27. Vaccination not accepted by peers
28. Pressure from family to not get vaccinated
29. Pressure from friends to not get vaccinated
30. Other p40v1) Which?______________________________________________
31. How far is your home from the vaccination center? _______ kilometers

**Thank you very much for participating in this survey.**

**The survey will supplemented with information on medical record of the patient:**

1. In the participant medical record is the CLAP form?

Yes;  No;  It was not possible to review the record.

If yes, (p42a) Is recorded the date on which it received the vaccine against seasonal influenza?

Yes;  No.

If not, (p42b) What were the reasons why have not access to the CLAP form?

We were not allowed access to the file

Although we were allowed access to the file, postpartum women did not take the cardo r CLAP fform to the hospital.

Other reason (p42b1) Which? _____________________________________

1. In the medical record of the participant is recorded some of the following chronic diseases:

| **Disease** | **Yes** | **No** | **No data** |  | **Disease** | **Yes** | **No** | **No data** |
| --- | --- | --- | --- | --- | --- | --- | --- | --- |
| 1. Chronic heart disease |  |  |  |  | 1. diabetes Mellitus |  |  |  |
| 1. Asthma |  |  |  |  | 1. chronic renal disease |  |  |  |
| 1. Bronchitis |  |  |  |  | 1. Immunosuppression (including HIV) |  |  |  |
| 1. COPD |  |  |  |  | 1. Cancer |  |  |  |
| 1. Cystic fibrosis |  |  |  |  | 1. cerebrovascular disease |  |  |  |

1. Gestational age at birth: ______ weeks (calculated by the system).
2. Gestational age at birth on medical record? _________weeks. (If no date last rule in the file)
3. Gestational age at ultrasound: _______weeks.
4. Ultrasound date _____ / _____ / _______ (dd / mm / yyyy).
