# Supplementary material for: Knowledge, attitudes, and practices of seasonal influenza vaccination in postpartum women, Honduras
Source: PLoS One. 2021 Feb 11;16(2):e0246385. doi: 10.1371/journal.pone.0246385 (PMC7877664; doi:10.1371/journal.pone.0246385)
Supplement: S1 File — (DOCX) [file pone.0246385.s003.docx]

**S1 File. Equation used to obtain sample sizes for survey of postpartum women**

Schaeffer R, Mendenhall W, Ott L. Elementary Survey Sampling, Boston. Massachusetts: PWS-Kent Publishing Company. 1990).

$$\boldsymbol{n=deff\times}\frac{\boldsymbol{N}\boldsymbol{Z}_{\boldsymbol{1-\alpha/2}}^{\boldsymbol{2}}\boldsymbol{P(1-P)}}{\left( \boldsymbol{N-1} \right)\boldsymbol{d}^{\boldsymbol{2}}\boldsymbol{+}\boldsymbol{Z}_{\boldsymbol{1-\alpha/2}}^{\boldsymbol{2}}\boldsymbol{P(1-P)}}$$

Where

*N* = population size, $Z_{1-\alpha/2}$ = Quantile of a variable with standard normal distribution

$100\left( 1-\alpha\right)\%=$ Confidence level

*P* = Expected proportion in the population.

*d* = Absolute accuracy required

$deff=$ Design effect
